# Deposition of Human-Serum-Albumin-Functionalized Spheroidal Particles on Abiotic Surfaces: Reference Kinetic Results for Bioparticles

**DOI:** 10.3390/molecules29143405

**Published:** 2024-07-20

**Authors:** Małgorzata Nattich-Rak, Marta Sadowska, Zbigniew Adamczyk, Teresa Basinska, Damian Mickiewicz, Mariusz Gadzinowski

**Affiliations:** 1 Jerzy Haber Institute of Catalysis and Surface Chemistry Polish Academy of Sciences, Niezapominajek 8, 30-239 Cracow, Poland; marta.sadowska@ikifp.edu.pl; 2 Centre of Molecular and Macromolecular Studies, Polish Academy of Sciences, Henryka Sienkiewicza 112, 90-363 Lodz, Poland; teresa.basinska@cbmm.lodz.pl (T.B.); damian.mickiewicz@cbmm.lodz.pl (D.M.); mariusz.gadzinowski@cbmm.lodz.pl (M.G.)

**Keywords:** albumin coronas on particles, bacteria deposition, deposition of albumin polymer particle conjugates, HSA-functionalized particles, spheroidal polymer particles, quartz microbalance measurements, zeta potential of albumin corona

## Abstract

Human serum albumin (HSA) corona formation on polymer microparticles of a spheroidal shape was studied using dynamic light scattering and Laser Doppler Velocimetry (LDV). Physicochemical characteristics of the albumin comprising the zeta potential and the isoelectric point were determined as a function of pH for various ionic strengths. Analogous characteristics of the polymer particles were analyzed. The adsorption of albumin on the particles was in situ monitored by LDV. The stability of the HSA-functionalized particle suspensions under various pHs and their electrokinetic properties were also determined. The deposition kinetics of the particles on mica, silica and gold sensors were investigated by optical microscopy, AFM and quartz microbalance (QCM) under diffusion and flow conditions. The obtained results were interpreted in terms of the random sequential adsorption model that allowed to estimate the range of applicability of QCM for determining the deposition kinetics of viruses and bacteria at abiotic surfaces.

## 1. Introduction

The adsorption of proteins on nano- and micro-sized particles is important for biosensing, enzymatic catalysis, bioreactors and immunological assays aimed at the detection of viral or bacterial infections, for example SARS-CoV-2, *Salmonella* and *E. coli* [1,2,3,4]. 

Given its essential significance, the adsorption of proteins on nanoparticles, which is referred to as corona formation, was extensively investigated both for single molecule systems and for mixtures [5,6,7,8,9,10,11,12,13,14,15,16,17,18,19,20,21,22,23,24,25,26,27]. However, a quantitative interpretation of the obtained results was hindered because of the reversibility of adsorption and the tendency of the functionalized particles to aggregate.

More reliable experiments can be conducted for particles above 100 nm (microparticles) regarding functionalization with antibodies [1,2,3] or blood serum proteins such as fibrinogen or HSA [28,29] because they form more stable suspensions. Thanks to this advantage, the corona formation at microparticles can be effectively monitored using electrophoretic mobility measurements and quantitatively interpreted in terms of theoretical approaches [30]. 

However, no results have been reported for the interesting case of protein corona formation on anisotropic polymer particles. Such functionalized particles can mimic the behavior of viruses composed of a core part containing RNA or DNA genome encapsulated by a hydrophobic membrane stabilized by various proteins [31,32,33,34,35]. Additionally, in the case of coronaviruses, such as SARS-CoV-2, spike proteins are flexibly incorporated into the membrane [36,37,38,39,40], enabling virus attachment to various bio-receptors and to abiotic surfaces. This process plays an essential role in virus inactivation and removal by filtration comprising various kinds of masks. 

Because of basic significance, the attachment of viruses and bacteria to various surfaces was often investigated by the quartz crystal microbalance (QCM) technique, enabling sensitive, real-time kinetic measurements under diffusion and flow conditions. For example, interesting studies based on QCM were reported for bacteria strains exhibiting a spheroidal shape [41], inter alia, *E. coli*, *Salmonella* [42,43,44] and soil bacteria [45]. Unfortunately, no attempt was undertaken to quantify the obtained results and to elucidate physical mechanisms of bacteria attachment. One can expect that the scientific impact of such tedious experiments could be enhanced if appropriate reference results obtained for well-characterized particles and sensors were available. 

It is postulated in this work that polymer particles functionalized in a controlled way by the adsorption of protein coronas can be used as reliable reference systems for acquiring essential information about mechanisms of virus and bacteria deposition on abiotic surfaces. In this work, attention is focused on the spheroidal polymer microparticle and human serum albumin (HSA) conjugates, which have not been studied previously in the literature. The main advantage of albumin is that its basic physicochemical properties such as molar mass, secondary structure, the size and the shape of the molecule are known [29,46,47]. Additionally, HSA forms stable solutions in electrolyte solutions of various pHs and ionic strengths. 

The adsorption mechanism of albumin on microparticles of a spherical shape was theoretically studied applying coarse-grained, Monte Carlo-type approaches [29]. These data can be exploited for a proper interpretation of the albumin corona formation investigations for spheroidal particles performed in this work. The progress of HSA adsorption is monitored under in situ conditions by Laser Doppler Velocimetry (LDV), yielding the electrophoretic mobility of the functionalized particles, which is converted to the zeta potential and interpreted in terms of a theoretical model. Subsequently, deposition of the particles on abiotic surfaces such as mica (used as a reference), silica and gold sensors is thoroughly investigated, which represents the main goal of this work. 

Because of the use of complementary theoretical and experimental methods, the obtained results furnished reliable information about mechanism of bioparticle deposition on solid/electrolyte interfaces, and about the range of applicability of the QCM method for such kinetic measurements. 

## 2. Results and Discussion

### 2.1. Physicochemical Characteristics of Albumin and Polymer Particles 

In order to facilitate a quantitative interpretation of the corona formation and particle deposition experiments, physicochemical characteristics of HSA and the polymer particle suspensions were acquired applying the above-mentioned techniques. 

The diffusion coefficient of HSA molecules at a pH range from 3.5 to 5 and a NaCl concentration of 10 to 30 mM directly measured by DLS was equal to 6.3 ± 0.3 × 10^−7^ cm^2^ s^−1^. This corresponds to the hydrodynamic diameter calculated from the Stokes–Einstein relationship, equal to 7.9 ± 0.4 nm. These values are similar to those previously reported in Reference [29], indicating that the HSA solutions were stable under these physicochemical conditions. 

**Figure 1:**
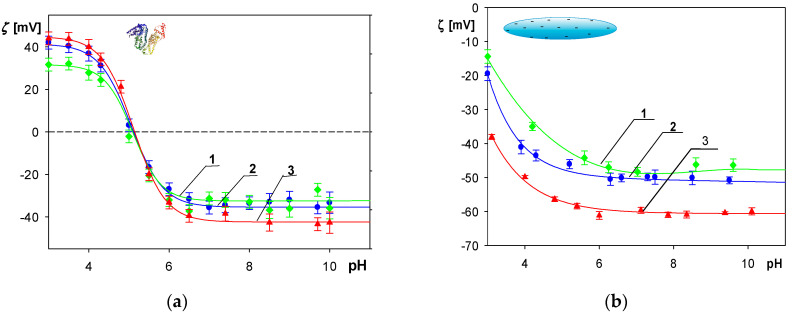
(**a**) Dependence of the zeta potential calculated from the Henry formula of HSA molecules on pH: 1. (▲) 30 mM NaCl; 2. (●) 10 mM NaCl; 3. (♦) 1 mM NaCl. (**b**) Dependence of the zeta potential of the spheroidal particles calculated using the Ohshima formula on pH: 1. (♦) 30 mM NaCl; 2. (●) 10 mM NaCl; 3. (▲) 1 mM NaCl. The lines represent a guide for the eyes.

The dependencies of the albumin molecule zeta potential on pH for NaCl concentrations of 1, 10 and 30 mM are shown in Figure 1a. As can be seen, at smaller pHs, the zeta potential was positive for all NaCl concentrations, attaining 36 ± 2 mV at pH 4 in 10 mM NaCl. However, for larger pHs it rapidly decreased, changing sign at pH 5. At pH 7.4 and 10 mM NaCl, the zeta potential attained a negative value of −36 ± 2 mV.

Physicochemical characteristics of the polymer particles were also analyzed. The dependence of their zeta potential on pH, calculated using the Ohshima formula for NaCl concentrations of 1, 10 and 30 mM, are shown in Figure 1b. The zeta potential was negative for the entire range of pHs assuming −40 ± 2 mV and −50 ± 3 mV at pH 4 and 7.4, respectively, for 10 mM NaCl. 

**Figure 2:**
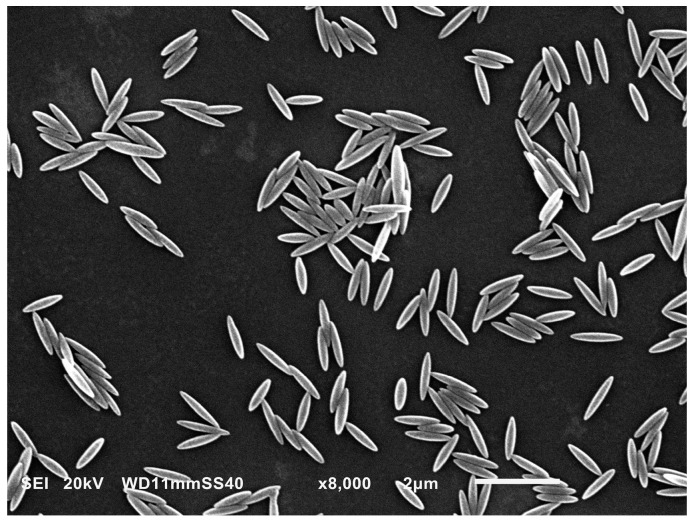
The SEM micrograph of the P(S/PGL) particle layer.

On the other hand, the diffusion coefficient of the particles determined by DLS was practically independent of pH and equal to 1.2 × 10^−8^ cm^2^ s^−1^ for the NaCl concentration of 10 mM. This corresponds to the hydrodynamic diameter of 400 ± 20 nm, calculated from the Stokes–Einstein formula. It should be mentioned that the hydrodynamic diameter alone, defined as the size of an equivalent sphere having the same diffusion coefficient, does not furnish information about the shape and true dimensions of the particles. These parameters were derived from scanning electron microscopy (SEM) micrographs, see Figure 2, as an average taken from ca. 100 particles. Thus, the particle dimensions were 1100 ± 50 × 220 ± 20 × 220 ± 20 nm, with the size distribution dispersity of ca. 5%. It was also confirmed that their shape can be approximated by a prolate spheroid with a longer to shorter axis ratio λ= *a*/*b* of 5 and a cross-section area in the side-on orientation equal to 0.19 mµ^2^. 

### 2.2. Formation of Albumin Corona on Polymer Particles

Functionalization of the spheroidal particles by the adsorption of HSA was carried out according to the procedure described in Ref. [29], where the changes in the electrophoretic mobility were monitored in situ by the LDV technique. The electrophoretic mobility of the particles acquired as a function of the initial albumin concentration was converted to the zeta potential and interpreted in terms of the electrokinetic model. It is worth mentioning that the time of corona formation at the polymer particles is very short compared to the adsorption at planar substrates and does not depend on the protein concentration. Using the formula derived in Reference [48] and the parameters pertinent to our measurements—the bulk particle concentration after mixing of 50 mg L^−1^, *d_H_* = 400 nm and the protein diffusion coefficient of 6.3 × 10^−7^ cm^2^ s^−1^—one can calculate that the corona formation time was equal to ca 0.1 s, which is considerably shorter than the experimental incubation time. 

**Figure 3:**
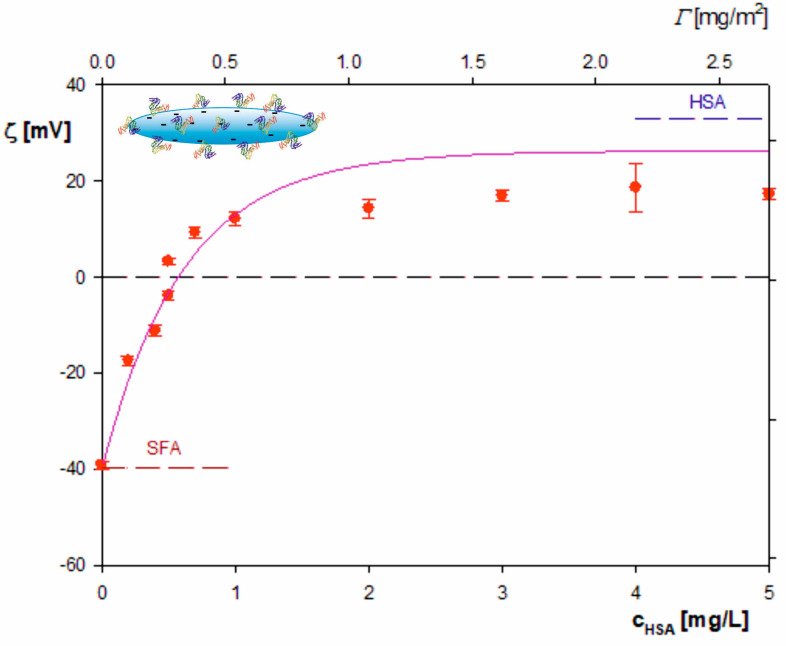
Dependence of the zeta potential of the polymer particle on the initial HSA concentration in the suspension *c_p_*; (●) experimental results derived from the LDV measurements of lower horizontal axis; experimental conditions: 10 mM NaCl, pH 4, initial particle concentration 100 mg L^−1^. The upper horizontal axis shows the nominal protein coverage calculated from Equation (6). The solid red line shows the theoretical results calculated from Equations (7) and (8), and the dashed/dotted horizontal line shows the linear fit of the experimental data for larger protein concentrations.

In Figure 3, the dependence of the zeta potential of particles on the initial concentration of HSA in the suspension acquired for 10 mM NaCl and pH 4 is shown. As can be seen, the initially negative zeta potential of the particles rapidly increased with the albumin concentration (denoted by *c_p_*) and became positive for *c_p_* larger than 0.5 mg L^−1^. For still larger concentrations of albumin, the zeta potential attained a plateau value of 15 mV, which was markedly lower than the bulk zeta potential of HSA molecules equal to 36 mV. 

One can observe that for the initial albumin concentration above 1 mg L^−1^, a saturated monolayer is formed on the polymer particles. The mass coverage of the albumin layer can be calculated with the following formula:

(1) $$\Gamma =\frac{c_{p}v_{p}}{S_{pol}}$$

where Γ is the protein mass coverage, conveniently expressed as mg m^−2^, $v_{p}$ is the protein solution volume and $S_{pol}$ is the net surface area of the particles given by

(2) $$S_{pol}=\frac{c_{pol}v_{pol}}{m_{1}}S_{1}$$

where $v_{pol}$ is the particle volume, *c_pol_* is the particle suspension concentration and $S_{1}$, $m_{1}$ are the surface area and mass of a single particle, respectively. 

Consider that for spheroids

(3) $$\begin{array}{l} m_{1}=\frac{4}{3}\pi b^{3}\lambda \rho_{pol}\\ S_{1}=4\pi b^{2}F_{s}(\lambda ) \end{array}$$

where $\rho_{pol}$ is the polymer particle density and $F_{s}(\lambda )$ is the correction function of the axis ratio parameter accounting for the increase in the specific surface area compared to spherical particles, which is given by

(4) $$F_{s}(\lambda )=\frac{1}{2}\left(1+\frac{\lambda^{2}}{{\left(\lambda^{2}-1\right)}^{1/2}}\arctan{\left(\lambda^{2}-1\right)}^{1/2}\right)$$

Considering Equations (2) and (3), one obtains the following formula for the mass average of albumin on the spheroidal particles:

(5) $$\Gamma ={F_{s}}^{-1}(\lambda )\lambda \left(\frac{\rho_{pol}b}{3}\right)\frac{c_{p}v_{p}}{c_{pol}v_{pol}}$$

For spheres, where λ = 1, $F_{s}(\lambda )=1$, for equal volumes of the protein and the particles, Equation (5) simplifies to the usual form [29,49]:

(6) $$\Gamma =\left(\frac{\rho_{pol}d_{p}}{6}\right)\frac{c_{p}}{c_{pol}}$$

where $d_{p}$ is the spherical particle diameter.

Considering the following parameters pertinent to the corona formation experiments, *c_pol_* = 100 mg L^−1^, $v_{p}=v_{pol}$, *b* = 110 nm, λ = 5 and $\rho_{pol}$ = 1.06 g cm^−3^, one can calculate from Equations (4) and (5) that the monolayer coverage of albumin on the particles was equal to ca. 0.5 mg m^−2^ for *c_b_* = 1 mg L^−1^. 

In order to obtain a more precise estimation of the monolayer coverage, the results shown in Figure 3 were interpreted in terms of the general electrokinetic model developed in References [30,48] using the following formula for the zeta potential of polymer particles covered by a protein layer, denoted by ζ:

(7) $$\zeta (\Theta )=F_{i}(\Theta )\zeta_{i}+F_{p}(\Theta )\zeta_{p}$$

where ζ is the zeta potential of the particles with the protein corona, Θ is the dimensionless protein coverage, *ζ_i_* is the zeta potential of the polymer particles, *ζ_p_* is the zeta potential of the protein in the bulk, and $F_{i}(\Theta ),\;F_{p}(\Theta )$ are the dimensionless functions. The *F_i_* function describes the damping of the flow near the particle surface by the adsorbed molecule layer, and the *F_p_* function characterizes the contribution to the zeta potential stemming from the electric double layer surrounding the protein molecules. These functions were calculated in Reference [30] by applying the multipole expansion method. The dimensionless coverage occurring in Equation (7) is connected with the mass coverage via the constitutive dependence:

(8) $$\Theta =S_{g}\left(\frac{{Ν}_{Av}}{M_{w}}\right)\Gamma$$

where *S_g_* is the characteristic cross-section area of the albumin molecule, ${Ν}_{Av}$ is the Avogadro number and $M_{w}$ is the molar mass of albumin, equal to 66,400 g mol^−1^. 

The theoretical results calculated from Equations (7) and (8) adequately reflected the experimental data for $c_{p}$ smaller than 1.3 mg L^−1^ (see Figure 3), whereas at larger protein concentrations the particle zeta potential attained a plateau value of 15 mV (this is depicted as the dashed horizontal line). Using this limiting concentration, one can calculate from Equation (8) that the mass coverage of the protein corona was equal to 0.65 mg m^−2^. 

It is interesting to mention that an identical value was previously determined for a silica sensor using the OWLS method [50], in Reference [51] the value of 0.6 mg m^−2^ was obtained by optical reflectometry and in Reference [29] one reported 0.7 mg m^−2^ for the recombinant HSA layer on a negatively charged polystyrene latex particle with a diameter of 800 nm. 

**Figure 4:**
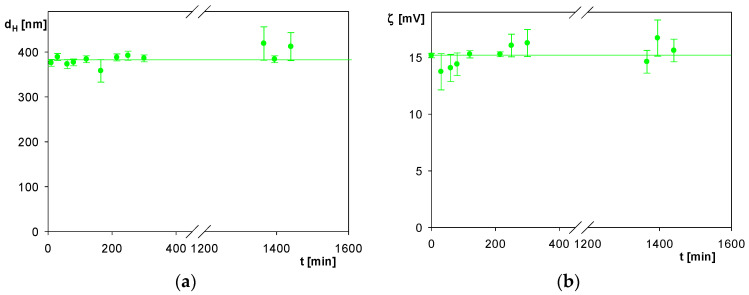
(**a**) Dependence of the hydrodynamic diameter of the SHSA polymer particles on the storage time: pH 4, 10 mM NaCl. The solid line shows the average value of the hydrodynamic diameter equal to 390 nm. (**b**) Dependence of the zeta potential of the particles on the storage time: pH 4, 10 mM NaCl. The solid line shows the average value of the zeta potential of 15 mV.

In the next series of experiments, the stability of the spheroidal particles functionalized by the albumin corona, hereafter referred to as SHSA, was determined. The results, shown in Figure 4 (for pH 4, 10 mM NaCl), were expressed as the dependence of the particle hydrodynamic diameter and the zeta potential on the storage time. As can be seen, the changes in the hydrodynamic diameter and the zeta potential were negligible for the storage time up to 1500 min (25h), which confirms an adequate stability of their suspensions. 

**Figure 5:**
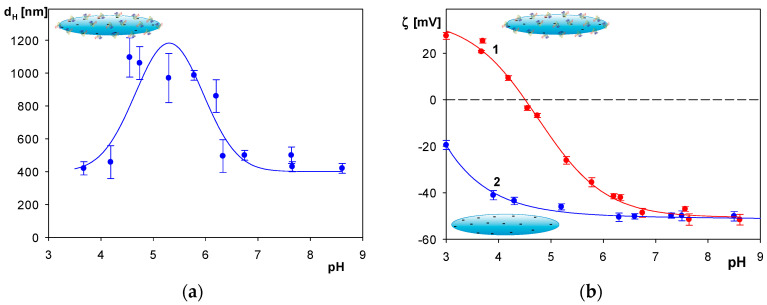
(**a**) Hydrodynamic diameter of the SHSA polymer particles vs. pH, 10 mM NaCl; the lines represent guides for the eyes. (**b**) (●) Zeta potential of the SHSA particles vs. pH; (●) zeta potential of the bare polymer particles vs. pH: 10 mM NaCl. The solid lines 1, 2 are guides for the eyes.

It should be mentioned, however, that for the pH within the range of 4.5 to 6.5, the hydrodynamic diameter of the particles markedly increased, attaining a maximum value of ca. 1100 nm (see Figure 5a). This increase is most likely caused by a reversible association of the SHSA particles under end-on orientations in fibrous-like structures. This hypothesis is supported by the dependence of the zeta potential of the SHSA particles on pH (see Figure 5b). As can be seen, the zeta potential of the particles abruptly decreased with pH and vanished at pH 4.7, which can be treated as their isoelectric point. Such a low zeta potential value promoted a reversible particle association at a pH around 5. At a pH larger than 7, the zeta potential of the SHSA particles approached the zeta potential of the bare particles (marked as curve 2 in Figure 5b).

### 2.3. Deposition of the SHSA Particles on Abiotic Surfaces 

**Figure 6:**
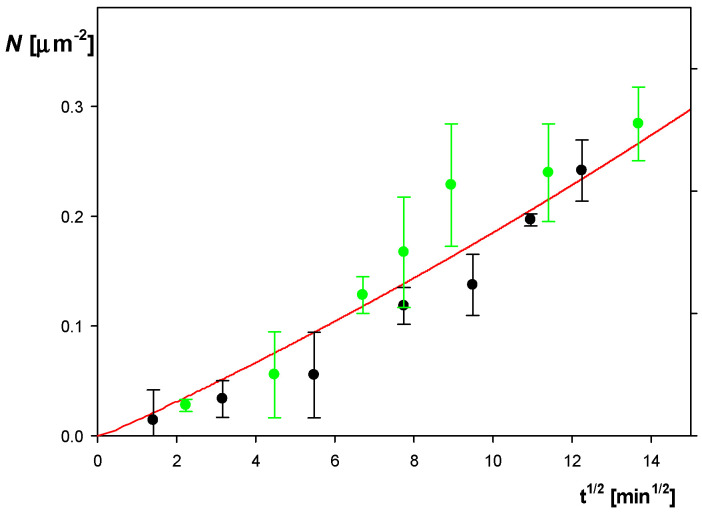
SHSA particle deposition kinetics on bare mica under diffusion presented as the dependence of its surface concentration *N* on *t*^1/2^ [min^1/2^], corona coverage 0.65 mg m^−2^, pH 4, 10 mM NaCl, particle bulk concentration 50 mg L^−1^. The green points represent the experimental results obtained by in situ optical microscopy. The inset shows the image of the particle layer characterized by *N* = 0.12 µm^−2^. The black points show the reference results obtained for the bare spheroid deposition on PAH-functionalized mica. The solid red line shows the theoretical results derived from the random sequential adsorption (RSA) model.

The deposition kinetics of the SHSA particles (bulk concentration 50 mg L^−1^, pH 4, 10 mM NaCl) on bare mica under diffusion is shown in Figure 6. As can be seen, the particle surface concentration (directly determined by the optical microscope enumeration technique) linearly increased with the square root of the deposition time. This behavior was adequately interpreted in terms of the theoretical results derived from the hybrid random sequential adsorption (RSA) approach [30,48], depicted as the solid red line in Figure 6. 

The results shown in Figure 6 confirm that the theoretical predictions calculated from the RSA model agree with the experimental data for *t*^1/2^ up to 14 min^1/2^ (ca. 200 min). This fact enables us to conclude that the particle deposition kinetics attained the maximum value pertinent to barrier-less transport conditions [48]. However, it should be mentioned that such kinetic experiments are tedious to perform because they require an image analysis of the deposited particle layers acquired at discrete time intervals. Hence, they are not recommended for routine measurements but can rather serve as useful reference data for the interpretation of experiments performed by other techniques, such as the quartz microbalance (QCM) technique, where the acquired signal cannot be directly related to the real particle coverage. On the other hand, the essential advantage of the QCM technique is that it yields real-time, quasi-continuous signals that are the sensor oscillation frequency and dissipation changes, which can be related to real coverage upon a proper calibration [52,53,54,55]. Usually, the QCM kinetic measurements are carried out under flow conditions in order to accelerate the experimental run. However, this markedly increases the consumption of the particles, which is disadvantageous for expensive solutes such as proteins, virus or bacteria or functionalized spheroidal particles produced in a tedious synthesis. Therefore, in this work, the measurements were predominantly carried out under diffusion transport conditions that manifold reduced the consumption of SHSA particles. Primarily, in these experiments the dependence of the frequency shift on the deposition time was recorded for various overtones $n_{0}$ (1 to 11 in our case). Then, the apparent QCM coverage, $\Gamma_{QCM}$, was calculated from the commonly used formula often referred to as the Sauerbrey equation [55]:

(9) $$\Gamma_{QCM}=C_{s}(-\Delta f/n_{0})$$

where Δ*f* is the frequency shift and *C_s_* is the Sauerbrey constant equal to 0.177 mg m^−2^ Hz^−1^ for the 5 MHz AT cut quartz sensor [55]. 

**Figure 7:**
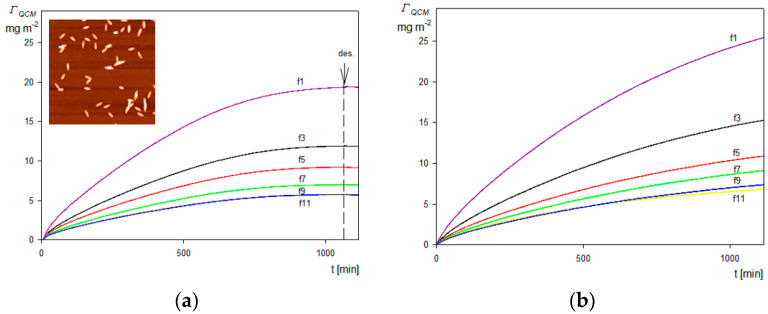
(**a**) Kinetics of SHSA particle deposition on the silica sensor under diffusion derived from QCM measurements: pH 4, 10 mM NaCl, *c_b_* = 50 mg L^−1^. The solid lines show the experimental data derived from Equation (9) for the various overtones (1 to 11); the arrow and the dashed vertical line show the beginning of the desorption run. The inset shows the image of the particle layer at the sensor acquired by AFM. (**b**) Reference results for the bare spheroid deposition at PAH-modified silica sensor: pH 4, 10 mM NaCl, *c_b_* = 50 mg L^−1^. The solid lines show the experimental data derived from Equation (9) for various overtones.

For a typical kinetic run recorded at pH 4, 10 mM NaCl, the bulk SHSA particle concentration of 50 mg L^−1^ and the silica sensor is shown in Figure 7a as the dependence of the QCM coverage calculated from Equation (9) on the time. In the inset to this figure, the AFM image of the particle layer on the senor is presented. As can be seen, the particles are deposited under the side-on orientation, as assumed by performing the theoretical interpretation of their deposition kinetics. For comparison, in Figure 7b, analogous results for the bare spheroidal particles without an HSA corona deposited on a PAH-functionalized sensor are also presented. 

As shown in Figure 7a, the particle coverage abruptly increased with time and attained after a 1100 min plateau values of 20 and 5 mg m^−2^ for the first and the eleventh overtone, respectively. Interestingly, the change in the particle coverage was negligible upon switching to the pure electrolyte flow (shown as the arrow and the dashed line), which can be interpreted as negligible desorption of particles. Analogous results were obtained in the case of the bare spheroidal particle deposition on the silica sensor functionalized by the PAH macro-ion according to the procedure described in Reference [56], see Figure 7b. This similarity of the deposition kinetics indicates that the basic mechanism of the bare and functionalized spheroids was the same. It should be mentioned that such a significant decrease in the QCM coverage calculated using Equation (9) with the overtone number was also observed in deposition kinetic experiments carried out for nano- and microparticles [55,57,58] as well as for viruses [33,35]. In the case of particles forming a stiff contact with the sensor, this effect can be attributed to hydrodynamic forces acting on the particle layer whose relative significance, compared to the inertia force, decreases with the oscillation frequency, i.e., the overtone number [57]. 

For larger particles, especially for spheroids, the hydrodynamic slip effect plays a decisive role, also inducing significant differences in the QCM coverage predicted from Equation (9) for various overtones [56]. This indicates that the interpretation of virus and bacteria deposition kinetics investigated by QCM can be rather ambiguous without considering the adequate theoretical background developed in Reference [56]. It was shown that the under the hydrodynamic slip regime, a more appropriate transformation of the frequency changes to obtain particle coverage has the following form:

(10) $$\Gamma_{QCM}=\alpha C_{s}(-\Delta f/n_{0}^{1/2})$$

where α is the dimensionless correction factor of the order of unity, mainly depending on the sensor roughness.

The exact value of α can be determined from the solution of the diffusion equation with the boundary condition at the sensor surface derived from the random sequential adsorption (RSA) approach [56], which was carried out without introducing any empirical parameters.

**Figure 8:**
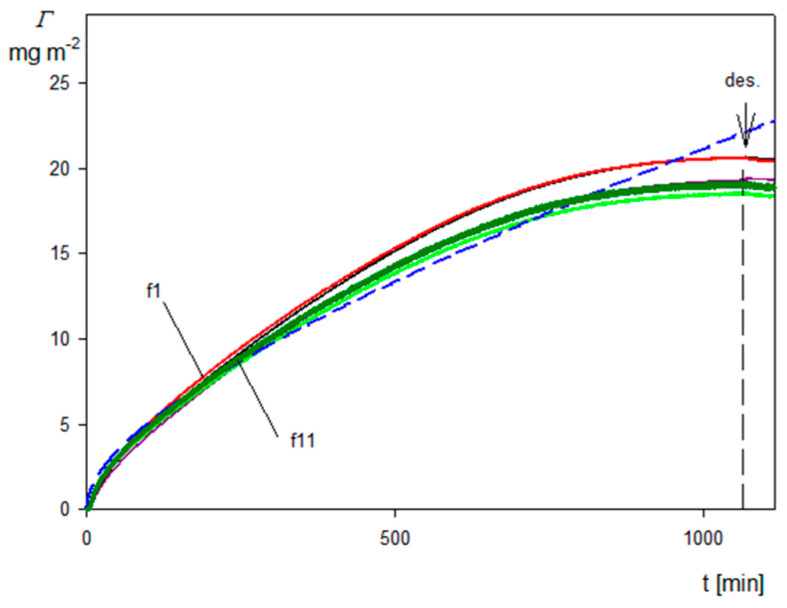
Kinetics of SHSA particle deposition on silica sensor under diffusion: pH 4, 10 mM NaCl, *c_b_* = 50 mg L^−1^, the arrow and the dashed vertical line show the beginning of the desorption run. The solid lines represent the QCM coverage calculated from Equation (10) for various overtones, the green solid line shows the kinetic averaged over all overtones, and the dashed blue line shows the theoretical results calculated from the RSA model.

**Figure 9:**
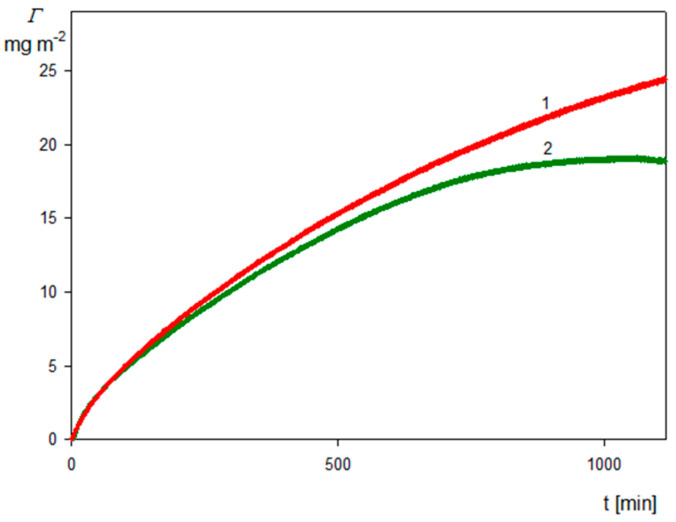
Comparison of SHSA particle deposition on different sensors under diffusion: pH 4, 10 mM NaCl, *c_b_* = 50 mg L^−1^. The solid red line (1) shows the kinetics calculated using Equation (10) and averaged over the overtones for the gold/PAH sensor, and the green line (2) shows the corresponding kinetics for the silica sensor acquired under the same experimental conditions.

Kinetics of the SHSA particle deposition on the silica sensor expressed using the transformation defined by Equation (10) (with α equal unity) are shown in Figure 8. As can be seen, the dependencies of the coverage on the deposition time calculated using Equation (10) for various overtones almost coincide with each other. This confirms the utility of this transformation, because one does need to arbitrarily choose the overtone number. In order to increase the precision of the QCM measurements and decrease the noise ratio, one can also calculate the kinetics averaged over all overtones, which is shown as the solid green line in Figure 8. As can be seen, the overtone averaged kinetics reasonably agree with that derived from the RSA modeling (dashed blue line in Figure 8). A quantitative agreement for the deposition time below 800 min can be achieved assuming that the correction factor α is equal to 0.9. This indicates that the QCM results transformed according to the proposed method can be applied for comparative studies of particle deposition kinetics under various physicochemical conditions. A comparison of the SHSA particle deposition kinetics on the silica and the gold/PAH sensors acquired at pH 4, 10 mM NaCl using the above transformation is shown in Figure 9. It can be seen that the kinetic runs calculated from Equation (10) (averaged over the overtones) for the bare spheroids on the gold/PAH sensor and the SHSA particles on the bare silica were practically identical for the shorter deposition time. However, at longer times, the deposition of the SHSA particles was less effective, which was probably caused by a lower adhesion strength of the latter particles. 

**Figure 10:**
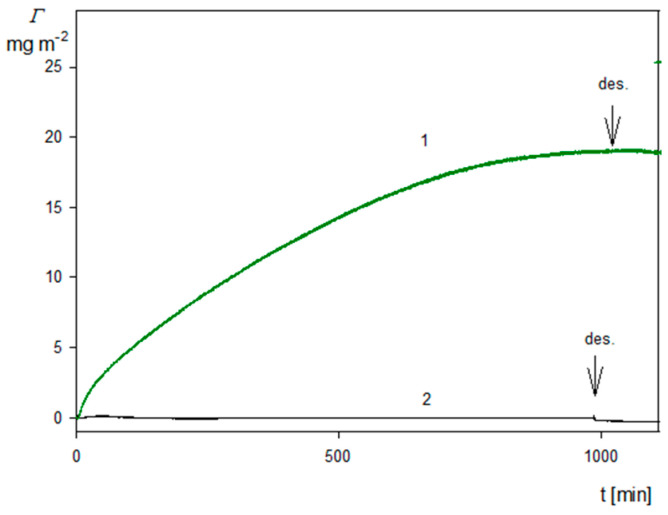
Kinetics of the SHSA particle deposition on the silica sensor under diffusion at different pHs calculated using Equation (10): 10 mM NaCl, *c_b_* = 50 mg L^−1^, the arrows show the beginning of the desorption run 1. Green line pH 4. 2. Black line pH 7.4.

The utility of the approach based on the above transformation is also confirmed by the results shown in Figure 10, where the SHSA deposition kinetics on the silica sensor at pH 4 and 7.4 are compared. One can observe that the deposition kinetics at pH 7.4 become practically negligible in comparison with pH 4, where they attained a maximum effectiveness governed by the bulk transport rate. It is worth mentioning that this behavior well correlates with the decrease in the particle zeta potential shown in Figure 5b, from 20 to −50 mV at pH 4 and 7.4, respectively, whereas the zeta potential of the silica sensor was negative at both pHs, equal to −40 and −50 mV, respectively. This behavior suggests that electrostatic interactions, which were predicted to be attractive at pH 4 and repulsive at pH 7.4, played a decisive role in the deposition of the SHSA particles. 

Analogous results were reported in References [33,34], presenting results of QCM investigations of the deposition of several bacteriophages on silica and gold sensors modified by self-assembled amine- and carboxyl-terminated layers (SAMs). Electrophoretic mobility measurements showed that the capsids exhibited a positive zeta potential for pH below 5 and negative otherwise, analogously as in our case for the polystyrene particles with the HSA corona. It was confirmed in References [33,34] that the deposition kinetics of the MS2 virus at the negatively charged carboxyl terminated self-assembled monolayer decreased with pH and vanished at pH 6.

**Figure 11:**
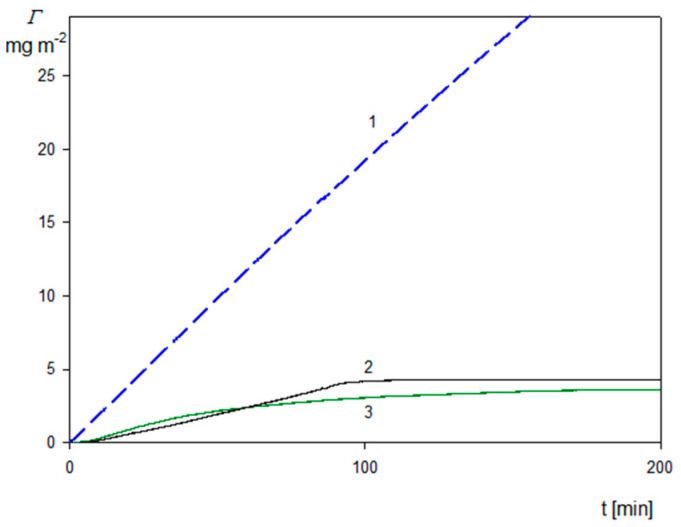
Kinetics of the SHSA particle deposition on the silica sensor under flow at different pHs calculated using Equation (10): 10 mM NaCl, *c_b_* = 50 mg L^−1^, flow rate 2.5 × 10^−3^ mL s^−1^. 1. The dashed blue line shows the kinetic for bare spheroidal particles on the silica/PAH sensor. 2. Black line pH 7.4. 3. Green line pH 4.

Additional QCM experiments were performed with the aim of determining the influence of flow on the deposition kinetics of the SHSA particles acquired under different pHs. The results presented in Figure 11 quite unexpectedly indicate that both at pH 4 and 7.4 the deposition rate of the SHSA particles was practically negligible compared to that observed for the bare spheroidal particles (shown as the dashed blue line in Figure 11). This effect can be interpreted as due to the flow-induced desorption of the particles from the sensor because of the appearance of hydrodynamic shearing forces. Apparently, these forces exceeded the particle/sensor adhesion force because of their low zeta potential, equal to 20 and −50 mV for pH 4 and 7.4, respectively. 

Therefore, the results presented in Figure 11 indicate that in the case of the functionalized particles bearing protein coronas, the QCM measurements carried out under diffusion conditions furnish more reliable results compared to those carried out under flow conditions. 

## 3. Materials and Methods 

The human serum albumin (nominal protein content of 99%) used in this work was supplied by Sigma-Aldrich (Merck, St. Louis, MO, USA) in the form of a lyophilized powder having a fatty acid content below 0.02%. Other chemical reagents, sodium chloride, sodium hydroxide, hydrochloric acid, sulfuric acid and hydrogen peroxide (Sigma Aldrich (Merck) St. Louis, MO, USA), were used without additional purification. Ultrapure water was obtained using the Milli-Q Elix&Simplicity 185 purification system from Millipore (Merck Group, Dermstadt, Germany). 

Poly(styrene/α-*tert*-butoxy-ω-vinylbenzyl-polyglycidol) (PS/PGL) spheroidal microparticles were obtained according to the method previously described in References [59,60]. This process consisted of three main steps: (i) synthesis of α-*tert*-butoxy-ω-vinylbenzyl-polyglycidol (PGL) macromonomer; (ii) synthesis of P(S/PGL) microspheres using styrene and a PGL macromonomer initiated with potassium persulfate, in water; (iii) preparation of spheroidal particles P(S/PGL) from the spherical ones applying the stretching of poly(vinyl alcohol) (PVA) films containing embedded P(S/PGL) microspheres and then the chemical modification of spheroidal particles to introduce a negative charge. The particle chemical composition was characterized by X-ray photoelectron spectroscopy (XPS), performed using the PHl 5000 VersaProbe—Scanning ESCA Microprobe (ULVAC-PHI, Japan/USA) instrument at a base pressure below 5 × 10^−9^ mbar. The size distribution and morphology of particles was characterized by scanning electron microscopy using a JEOL 5500LV apparatus (Akishima, Japan). 

Ruby mica supplied by Continental Trade, Poland, was used for the HSA adsorption kinetic measurements investigated by optical microscopy and atomic force microscopy (AFM). Thin sheets of mica were freshly cleaved before each experiment and used without any pretreatment. 

The bulk concentration of albumin in the stock solution, prepared by dissolving the powder under controlled pH and ionic strength, was spectrophotometrically determined using the procedure described in Reference [29]. The concentrated stock solution was diluted by a pure electrolyte of a fixed pH and ionic strength to a desired concentration before each experiment. 

The diffusion coefficients of the HSA molecules and polymer particles were determined by dynamic light scattering (DLS) using the Zetasizer Nano ZS (Malvern, UK). Respective hydrodynamic diameters were calculated from the Stokes–Einstein equation. 

The electrophoretic mobility of the functionalized spheroidal particles and HSA molecules was measured by the Laser Doppler Velocimetry (LDV) technique using the Zetasizer Nano ZS device. Using the electrophoretic mobility data, the corresponding zeta potentials were calculated using the Ohshima [61] and Henry [62] formulae, respectively. 

The optical microscopy measurements of the adsorption kinetics were conducted using the inverted microscope LABOPHOT-2 under dark-field illumination. The microscope was oriented horizontally with the objective axis perpendicular to the diffusion cell wall made of a mica sheet. This was advantageous because possible disturbances stemming from particle sedimentation were eliminated. 

Atomic force microscopy (AFM) measurements were carried out using the NT-MDT OLYMPUS IX71 device with the SMENA scanning head. The measurements were performed in semi-contact mode using silicon probes and polysilicon cantilevers HA-NC ETALON with resonance frequencies of 140 kHz +/− 10% or 235 kHz +/− 10%.

The QCM deposition kinetic experiments were performed according to the standard procedure described in References [52,53,54,55]. Initially, a stable baseline for the pure electrolyte (NaCl) at a fixed ionic strength and pH was obtained. Afterward, the particle suspension was flushed through the cell at a fixed flow rate. After a prescribed time, the pure electrolyte solution of the same pH and ionic strength was flushed in order to study particle desorption. The sensors with deposited particle layers were examined after completing the desorption run by ambient air AFM. The gold/quartz/silicon dioxide (SiO_2_) sensors used in the experiments were supplied by Q-Sense, Gothenburg, Sweden, whereas the bare gold sensors used in the experiments were supplied by QuartzPro, Jarfalla, Sweden. Both sensor types were characterized by a fundamental frequency of 5 MHz. They were cleaned before each experiment in a mixture of 95% sulfuric acid (H_2_SO_4_) and hydrogen peroxide (30%) in volume ratio of 3:2 for 10 min. Afterward, the sensor was rinsed by deionized water at 80 °C for 30 min and dried out in a stream of a nitrogen gas. The roughness of sensors was examined by semi-contact mode by AFM imaging carried out under ambient conditions. The root mean square (rms) roughness of the gold/silica and the bare gold sensors was equal to 1.0 ± 0.1 and 1.5 ± 0.2 nm, respectively. 

The pH of the protein solutions was adjusted in the range of 3 to 5 by the addition of HCl, whereas a pH of 7.4 was fixed by the PBS buffer, and larger pHs were adjusted by NaOH. 

The temperature of the experiments was fixed at 298 ± 0.1K.

## 4. Conclusions

The formation of albumin coronas on polymer particles of a spheroidal shape was effectively monitored by the LDV method and quantitatively interpreted in terms of the electrokinetic model. It was confirmed that the physicochemical properties of the particles comprising their size and zeta potential were stable for prolonged storage time. 

A useful procedure for an effective interpretation of the deposition kinetics results derived from QCM for the particle-bearing protein coronas was developed. It is based on Equation (10), which enabled transformation of the kinetic runs obtained for various overtones to one universal dependence. 

It was confirmed that the deposition kinetics of the particles under diffusion at pH 7.4 were negligible in comparison with pH 4, which correlated with the decrease in their zeta potential. This effect was interpreted as the indication of an electrostatic mechanism of particle deposition.

It was also shown that the QCM investigation carried out under diffusion rather than under flow conditions was more reliable. There are two major advantages of such measurements: (i) a significant decrease in the consumption of the expensive solutes; (ii) the elimination of the ortho-kinetic (induced by the flow) aggregation of the particles in the cell by hydrodynamic shearing forces. 

One can argue that the obtained results furnished reliable information about the deposition mechanism of the functionalized spheroidal particle and about the range of applicability of the QCM method for such kinetic measurements. These data can be used as useful references for the interpretation of bioparticle deposition at solid–electrolyte interfaces, especially on QCM sensors. 

## Data Availability

Data are available on request.
